# Review of Lyme Borreliosis in Africa—An Emerging Threat in Africa

**DOI:** 10.3390/biology13110897

**Published:** 2024-11-04

**Authors:** Nejib Doss, Aldo Morrone, Patrizia Forgione, Giusto Trevisan, Serena Bonin

**Affiliations:** 1 Department of Dermatology, Military Hospital of Tunis, Tunis 1008, Tunisia; nejib.doss@gmail.com; 2 IRCCS Dermatologic Institute San Gallicano, 00144 Rome, Italy; aldomorrone54@gmail.com; 3 Dermatology Unit, Lyme Disease Regional Center, 80134 Naples, Italy; patriziaforgione2@gmail.com; 4 Department of Medical Sciences, University of Trieste, 34149 Trieste, Italy; trevisan@units.it

**Keywords:** *Borreliae* Lyme Group, migratory birds, ticks, erythema migrans, neuroborreliosis, reservoirs

## Abstract

Lyme borreliosis (LB) is more common in the Northern Hemisphere. It is endemic mainly in North America, where the vectors are *Ixodes scapularis* and *Ixodes pacificus*, and in Eurasia, where the vectors are *Ixodes ricinus* and *Ixodes persulcatus*. Both tick-borne diseases and LB are influenced by climate change. Africa and South America are crossed by the equator and are situated in both the Northern and Southern Hemispheres. In Africa, the LB is present on the Mediterranean and the Indian Ocean coasts. *Borrelia lusitaniae* is prevalent in countries bordering the Mediterranean Sea, such as Tunisia, Morocco, Algeria, and Egypt. Ticks were detected in the *Ixodes Ricinus*, which are carried by migratory birds and the *Ixodes inopinatus* and captured by the *Psammodromus algirus* lizards. The *Borreliae* Lyme Group (LG) and, in particular, *Borrelia garinii*, have been reported in countries bordering the Indian Ocean, such as Kenya, Tanzania, and Mozambique, transported by migratory birds from North African countries, where the vector was identified as *Hyalomma rufipes* ticks. This review aims to document the presence of *Borreliae* LG and LB in Africa.

## Simple Summary

Although Lyme Borreliosis is endemic in countries of the Northern Hemisphere, climate change, migratory birds, and the availability of ticks have favored the survival of *Borreliae* Lyme Group in African countries bordering the Mediterranean Sea and the Indian Ocean.

## 1. Introduction

The *Borrelia* genus is divided into three groups, namely the Lyme Group (LG), the Echidna-Reptile Group (REPG), and the Relapsing Fever Group (RFG) [1].

Lyme borreliosis (LB) is a form of anthropozoonosis caused by the infection of *Borrelia burgdorferi* sensu lato (Bbsl) belonging to LG, usually transmitted by a hard tick of the genus *Ixodes*. Clinically, the first manifestation (present in 75% of cases) is an erythema that spreads around the tick bite (erythema migrans). Other clinical manifestations are migrating arthralgias, neurological manifestations, skin manifestations (with acrodermatitis chronica atrophicans observed in the late stage), and cardiac manifestations, in particular arrhythmia and pericarditis [2].

Currently, 24 species are included in *Borreliae* LG; to the 23 already reported [1], the most recent addition is *Borrelia maritima*, which was isolated in California [3]. Nine *Borreliae* LG are pathogenetic to humans. Of these, *B. afzelii* is mainly associated with skin manifestations, while *B. garinii* and *B. bavariensis* are associated with neuroborreliosis [4]. Lyme arthritis is preferentially related to *B. burgdorferi sensu strictu* (Bbls). [5]. Also, *B. spielmanii*, *B. valaisiana*, *B. bissettii*, *B. lusitaniae*, and *B. mayonii* are pathogenic to humans. Of these, *B. mayonii* causes spirochetemia [6]. *B. lusitaniae* was isolated in Portugal from human acrodermatitis chronica atrophicans lesions (strain PoHL1), and from *I. ricinus* ticks [7], and it has also been identified in lizards. The host-specific infection of these spirochaetes supports that they are specialized in the enzootic cycle, and through the migratory birds, they have crossed the Mediterranean Sea arriving in North Africa [8]. Vectors of *Borreliae* LG are usually hard ticks of the genus *Ixodes*, which prefer a cool–humid microclimate in spring and autumn, showing greater activity when the humidity is around 90%. Tick mortality, host-seeking activity, and the height and duration of ticks’ quests above ground are significantly impacted by low humidity exposure [9]. Therefore, the rainy climates favor their diffusion and reproduction whilst *Ixodes* ticks assume an endophilic life in summer with high temperatures [10]. The distribution of tick-borne pathogens is primarily associated with the density of ticks and with reservoir animals. In Europe, competent host reservoirs of *Borreliae* LG include common species of small and medium-sized rodents (*Apodemus flavicollis*, *Myodes glareolus*), as well as several species of birds (in particular passerines, such as *Turdus merula*) [11].

*Borreliae* LG can also be transmitted by cofeeding, which occurs when *Borrelia* is transferred between infected and naive vectors that feed in close spatiotemporal proximity to a host that has not yet developed a systemic infection [12].

Lyme borreliosis (LB) is mainly present in the Northern Hemisphere, mostly in North America [13] and in Europe [14], with a distribution that is closely related to *Ixodes* species, notably *Ixodes ricinus* in Europe, *I. persulcatus* in Asia and Eastern Europe, and *I. scapularis* and *I. pacificus* in the US. In 2022, 62,551 cases of Lyme disease were reported to the US Centers for Disease Control and Prevention (CDC) [15], representing 1.7 times the annual average (37,118 cases) for the period 2017–2019 [16]. However, according to the health insurance claims database, the annual incidence of Lyme disease diagnoses per 100,000 enrollees ranged from 49 to 88 in the period ranging from 2010 to 2018 in the US, which is about 6–8 times higher than that observed for cases reported through notifiable disease surveillance [17], which was about 476,00 cases/year [18]. In Europe, the population-weighted incidence rate of LB was estimated to be 22.04/100,000 person-years, with 200,000 new cases of LB per year in Western Europe [19]. In Italy, LB is widespread throughout the territory, but it is endemic in the Northern Alpine regions [20].

The spread of LB is closely related to the distribution of hard ticks, mainly of the *Ixodes* genus. These ticks are highly plastic and can adapt even to suboptimal environmental conditions. They prefer a humid habitat, which allows them to spread and develop rapidly. Consequently, these ticks are mainly present in the Northern Hemisphere, decreasing approaching the equator and in the Southern Hemisphere [21].

*Ixodes ricinus* is present in Europe and North Africa. Its great adaptability increases the risk area for infection by *Borreliae* LG and other tick-borne pathogens. The sheep tick, *I. ricinus*, has a low specificity that enables it to infest various host species, such as birds and mammals (including humans) [22]. LB is less frequent in the Southern Hemisphere [23], where the vector ticks can be of the genus *Ixodes* but also of the *Hyalomma*, *Amblyomma*, and *Riphicephalus* genera [24].

Africa and South America, crossed by the equator line, are represented in both hemispheres and are in contiguity with Europe and North America, respectively, where LB has a high incidence. The increase in cases of Lyme disease has been associated with the increase in reservoirs and the long summer seasons caused by climate change. In addition, the warming of temperatures has caused an expansion of *Ixodes* spp. ticks [25].

This study aims to highlight the different clinical, epidemiological, and management peculiarities of LB in the African continent.

## 2. Africa

In Africa, hard ticks belonging to the genera *Hyalomma*, *Ixodes*, *Amblyomma*, *Dermacentor*, *Haemaphysalis*, and *Rhipicephalus* can parasitize livestock, camels, horses, giraffes, and domestic animals (dogs). Some of these ticks damage livestock by transmitting infections, which can cause anemia and abortions, leading to a consequent reduction in food products (milk and meat) and significant economic losses [26]. Tick-borne diseases affecting livestock are particularly relevant for the livelihood of resource-poor farm communities, such as in sub-Saharan Africa [27]. Ticks can damage livestock merely by injecting the neurotoxins contained in their saliva. This has been reported for *Ixodes rubicundus*, which is responsible for the Karoo paralysis in sheep, goats, and young calves in South Africa [28], and for *Ixodes holocyclus* in Australia [29]. Of note, *Ixodes rubicundus* can survive in mountain and desertic environments [28].

In Africa, ticks transmit several pathogens, including *Borreliae*, which most often belong to the Relapsing Fever Group (RFG), where the vector is often a soft tick, usually of the genus *Ornithodoros* spp. or similar. The RFG *Borreliae* are transmitted vertically into the ticks, which can act as a reservoirs (*O. moubata*) and contribute to the maintenance and spread of the infection [30]. In LG *Borreliae*, the vertical transmission is negligible, and ticks should be infected by a reservoir host (rodents or lizards) to transmit LB [31]. RFG *Borreliae* can also be transmitted by hard ticks (Hard-Tick-Borne Relapsing Fever—HTBRF). Both *Borrelia miyamotoi* and *Borrelia theileri* are present in Africa [32].

*Borrelia miyamotoi* is transmitted by *Ixodes* spp. ticks, the same as LB, and can infect humans [33,34] and wildlife [35]. *Borrelia theileri*, transmitted by *Boophilus* (*Rhipicephalus*) *microplus* [36], infects domestic animals and livestock [37].

The distribution of Bbsl in Africa is not fully known and is closely related to migratory birds, ticks of *Ixodes* and *Hyalomma* genera, lizards as possible reservoirs, and climate change.

The migratory birds, infested by *Ixodes frontalis* and *Ixodes ricinus*, have probably led to the settlement of *Borrelia lusitaniae* from Portugal to North Africa, notably to Tunisia, Morocco, and Algeria [38] (see Table 1). *Ixodes ricinus* and *I. inopinatus* have been detected and characterized in the humid area of Northern Tunisia [39]. *I. Inopinatus*, in addition, seems to extend also to the sub-humid area [39] and is usually infesting the lizards [40]. Lizards are one of the least studied taxa, and in Africa, they play an important role in the enzootic cycle of the Bbsl, acting as reservoirs and amplifiers of these spirochetes [41].

Both *I. ricinus* and *I. inopinatus* can transmit *Borrelia burgdorferi* s.l.; however, *B. lusitaniae* is prevalent in North Africa, with genotypes supporting a population division separating samples from southern Portugal and Algeria from samples from northern Portugal and other European countries [42].

The other tick species that have been recovered and identified infesting small ruminants and wild animals (giraffes and buffaloes) [43] throughout Africa belong to the genera *Amblyomma*, *Rhipicephalus*, and *Hyalomma* [24]. Of these, *Hyalomma* ticks, including also *H. rufipes*, which tested the highest positivity for tick-borne pathogens, have been collected from sheep, camels, horses, goats, and dogs in Sudan [44]. *Hyalomma marginatum* infests mainly sheep and goats in North Africa and Ivory Coast [45], while *Hyalomma dromedarii*, parasitizing mainly camels, sheep, and goats, is found in Algeria, Ethiopia, Nigeria, and Sudan [46].

**Figure 1:**
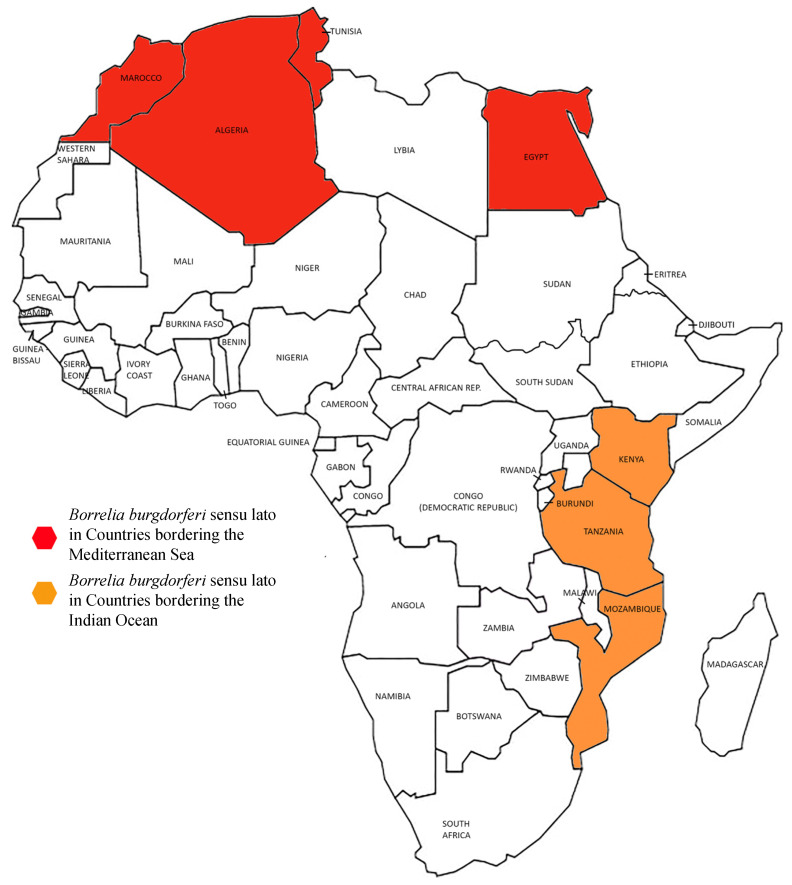
*Borreliae* LG distribution in Africa.

The presence of Bbls has been documented in ticks collected in North African countries, along the Mediterranean Sea (Algeria, Morocco, Tunisia, and Egypt), and in the countries bordering the Indian Ocean (Tanzania, Kenya, and Mozambique) (Figure 1), but infection in humans appears currently negligible [47].

The following paragraphs describe the documentation of LG *Borreliae* in detail for each of the abovementioned countries.

### 2.1. African Countries Along the Mediterranean Sea

In North African countries, a Mediterranean climate predominates in the coastal belt (Maghreb) from Morocco to Tunisia, with four seasons characterized by hot and dry summers and cool and wet winters, which is more suitable for the settlement of *Ixodes* spp. ticks and *Borreliae* LG. The climate becomes arid in the south (Sahara desert), extending from the Atlantic Ocean to the Red Sea. At the eastern edge of the Sahara flows the Nile River, which starts from the tributaries of Sudan Azzurro and Atbara and opens into the Mediterranean. In this area, the climate is tropical.

These climatic conditions and the unique range of geographic features support a wide diversity of tick species and tick-borne diseases in North Africa [48].

#### 2.1.1. Algeria

*Borrelia lusitaniae* (strain ID 3117) has been documented in *Ixodes inopinatus* ticks in Kabylie [42]. LB was detected in two horses (*Equus caballus*) by indirect immunofluorescence antibody and ELISA tests [49].

#### 2.1.2. Egypt

Egypt is positioned on a migratory route, the African–Eurasian way, for birds migrating between breeding sites in Eurasia and wintering sites in Africa, crossing the Mediterranean Sea. Migratory birds crossing Egypt are infested by several species and subspecies of ticks, including *Ixodes* spp. and *Hyalomma* spp.; this infection can occur during the migratory flight. Accordingly, Bbsl has been identified from *Hyalomma dromedarii* ticks infesting camels, and from *Rhipicephalus annulatus* from cattle [50]. Molecular analyses carried out in *Hyalomma dromedarii* and *H. marginatus* ticks and in the blood of infested camels in Cairo and Giza revealed *Borrelia afzelii* (LG) and *Borrelia miyamotoi* (RFG) [51]. Bbls were also detected in dogs, although at a relatively low rate both by molecular methods [52] and by serology [53].

Regarding human infection in Egypt, LB was documented in two farmers living in the Nile Delta [54]. Earlier, in 1995, four guys in El-Shatby reported skin lesions suggestive of erythema migrans and myo-arthralgia, and all of them tested positive for antibodies against *Borrelia burgdorferi* by ELISA [55].

#### 2.1.3. Mali

LB has not been documented in Mali [56]; however, its geographical location, bordering Algeria, suggests that its presence cannot be ruled out.

#### 2.1.4. Morocco

In 2002, Bbls was documented for the first time in Morocco with 26 pure culture isolates, of which 25 were *B. lusitaniae* and one was *B. burdorferi ss*. In ticks, *B. garinii* was also isolated [57]. Infection with LG *Borreliae*, confirmed by Western blot analysis and causing neuroborreliosis, was documented in a 23-year-old woman in Morocco [58].

#### 2.1.5. Tunisia

Lyme borreliosis is quite well documented in Tunisia. From 1988 to 1992, 23 cases of erythema migrans were detected in Sfax [59], and from 1992 to 1996, 29 cases of Lyme disease were identified by ELISA, each involving neurological and articular manifestations [60]. Bbsl infection was also investigated in ruminants, notably goats, sheep, cattle, and camels. Animals were infested by ticks only of the *Rhipicephalus* and *Hyalomma* genera. The prevalence of *B. burgdorferi s.l.* in those animals varied significantly according to the humidity of the localities and revealed a significantly higher rate of infection in goats that were located in humid areas than those located in sub-humid areas [61].

In 2014, the *Ixodes inopinatus* tick was identified in North Africa [40]. This tick is the main vector of *Borrelia lusitaniae* and parasitizes mostly lizards (*Psammodromus algirus*) and foxes, but also occasionally humans and other animals, including horses [62]. Isolates of *B. lusitaniae* (15/16) and *B. garinii* (1/16) were obtained from ticks, namely *Ixodes ricinus* and *I. inopinatus* [63], sharing the same geographic areas in Northern Tunisia [64].

It is more likely that *Psammodromus algirus* lizards could act as reservoirs for *B. lusitaniae*; 17% of ticks feeding on *P. algirus* indeed acquired *B. lusitaniae*, demonstrating the ability of the lizards to sustain *Borrelia* infection [65]. Also, the sand lizard, *Lacerta agilis*, is thought to be a reservoir of *B. lusitaniae.* Collar scale samples of the lizards tested PCR-positive for *B. lusitaniae*, and, in addition, some *I. ricinus* collected from the lizards were PCR-positive for Bbls [66].

### 2.2. African Countries Bordering the Indian Ocean

African countries bordering the Indian Ocean have tropical climates, which can facilitate the survival and spread of *Borreliae* LG.

#### 2.2.1. Kenya

Kenya has three different climates, with large regional climatic variations influenced by several factors, including altitude. There are two dry seasons: the first one runs from December to March and the second from July to October. The two rainy seasons come in between. The heaviest rains usually fall from mid-March to May.

Most studies carried out in Kenya have focused on the veterinary cases of tick-borne pathogens. Ticks collected from livestock from the major abattoirs in Nairobi and Mombasa revealed a 5% prevalence of infection from *B. burgdorferi* s.l. [67]. In addition, this study also showed that ticks of the genus *Rhipicephalus* and *Amblyomma* were the dominant carriers of zoonotic pathogens and that LG *Borreliae* were almost detected in *Rhipicephalus pulchellus* [67].

From 2009 to 2017, ticks were collected in various locations in Kenya from sympatric wild and domestic animals, and *B. garinii* DNA was detected in a *H. Rufipes* tick collected from a giraffe [68]. *B. garinii* is usually vectored by *Ixodes* ticks in Europe and Asia, but it has also been documented in North Africa, vectored by *I. ricinus* and *I. inopinatus* [40,47].

The picture of LB in human cases is incomplete in Kenya. In 2005, two men from the Rift Valley developed neuroborreliosis after a tick bite [69]. In a study investigating seroprevalence for different tick-borne pathogens in 1033 patients presenting with acute febrile illness in nine healthcare facilities from different ecological zones of Kenya, 21% tested positive for LB. In addition, exposure to LB was highest in the younger age group [70].

The presence of *Borreliae* LG in these countries is probably linked to the Europe–Africa–Europe two-way transport mechanism of the African ticks *Hyalomma rufipes*, which parasitizes migratory birds, such as *Acrocephalus schoenobaenus*, traveling from Africa to Southern Norway and Finland. This is not the first case where *Ixodes* spp., a vector of *Borrelia* LG, is replaced by *Hyalomma* spp.; previously, indeed, *B. lusitaniae* (strain PotiB1) was found in *H. marginatum* [71]. Long-distance exchange of tick-borne pathogens due to migratory birds is clearly the basis of the introduction of Bbls in Africa. It is possible that tick exchanges occur between birds migrating to Northern Europe and birds migrating to Africa [72]. This is also supported by the geographic distribution of the ticks: *Hyalomma* spp. is more represented in Africa, while *Ixodes* spp. is more represented in Europe.

#### 2.2.2. Mozambique

Mozambique has a tropical to subtropical climate. Rainfall distribution follows a north–south gradient, with more rainfall along the coast. The rainy season lasts from January to March. The south of Mozambique is generally drier.

The first autochthonous case of LB was documented in Mozambique in 1993 [73]. Some years later, a cross-sectional serological survey was carried out in Maputo for LB and leptospirosis, which revealed only some cases of seropositivity for LB, which was more likely attributed to cross-reactivity. Since no molecular characterizations were carried out, LB has been considered an unlikely occurrence in this country [74].

#### 2.2.3. Tanzania

Tanzania has a generally comfortable climate year-round, although there are significant regional variations. The tropical coast is quite hot and humid with heavy and reliable rainfall, especially during the rainy seasons, from mid-March to May and from November to mid-January.

Two studies in Tanzania investigated the presence of tick-borne pathogens in ticks that were collected from cattle and wild animals. In each tick, there was evidence of *Borreliae* LG [75,76].

Despite those findings, in Dar es Salaam, a pregnant woman developed erythema migrans after a tick bite. She also had arthralgia and tested positive for *Borrelia* on an ELISA test [77]. The same author reported other patients with clinical symptoms suggestive of LB and positive serology [77].

#### 2.2.4. Other African Countries

There are no reports on LB in the following African countries: Angola, Benin, Botswana, Burkina-Faso, Burundi, Cameroon, Cape Verde, Central African Republic, Chad, Congo Republic, Equatorial Guinea, Eritrea, Ethiopia, Gabon, Gambia, Ghana, Guinea, Ivory Coast, Lesotho Kingdom, Liberia, Libya, Madagascar, Malawi, Mauritania, Mauritius, Namibia, Niger, Nigeria, Rwanda, Senegal, Seychelles, Sierra Leone, Somalia, South Africa, Sudan, and Togo.

## 3. Discussion

Tick-borne diseases are one of the risks associated with global warming and climate change. Many studies predicted and already noted the expansion of tick habitats, both northern and southern [78,79]. This can determine the diffusion of tick-borne pathogens in areas that were previously not endemic. Climate variables, such as temperature and humidity, influence ticks’ survival and define their habitat areas [80]. The presence and spread of Bbls in Africa are closely related to:

- migratory birds;
- climate change;
- *Hyalomma* spp. ticks becoming Bbsl vectors;
- reservoirs other than small rodents, such as lizards;
- infection in humans, domestic animals, livestock, and wildlife.

### 3.1. Migratory Birds

The presence of *Borreliae* LG in North African countries is due to migratory birds infested by *Ixodes ricinus* and *I. frontalis*, acting as vectors for *Borreliae* LG, most commonly *Borrelia lusitaniae*, which, according to phylogenetic analysis, most likely originated in Serbia and spread later throughout Europe and North Africa. Many bird species travel annually across the Mediterranean Sea from their European breeding sites to their wintering sites in Africa, and then back again. Ticks infesting these birds are often transported in a two-way flow from Europe to North Africa via the Mediterranean Sea [48]. The analyses of the routes confirmed that the birds fall under the families *Sylviidae*, *Muscicapidae*, *Turdidae*, and *Acrocephaliidae* [81]. This trans-Mediterranean flow of ticks and their pathogens creates a dual threat to health in both Europe and North Africa, enabling some of these species to settle in new areas [82]. Scenarios regarding the future spatial distribution of *I. ricinus* already show overlaps between the North African region and Europe [83].

### 3.2. Hyalomma spp. Ticks as Vectors of Bbls 

In spring and autumn, migratory birds transport *Ixodes ricinus* with Bbsl from Europe to North Africa and *Acrocephalus schoenobaenus* birds transport *Hyalomma rufipes* from Africa to North European countries. The latter ticks can transport new infections in Europe, such as the Crimean-Congo hemorrhagic fever (CCHF), isolated from *H. marginatum* ticks carried by migratory birds *Iduna opaca*, *Phylloscopus trochilus*, and *Sylvia communis* from Morocco [79,84]. At the same time, those ticks can become infected by Bbsl, which they can serve as vectors for in African countries, as shown for di *B. lusitaniae* infecting *H. marginatum* and *B. garinii* infecting *H. rufipes* [8,71]. The mechanism allowing the transmission of *Borreliae* is the cofeeding of ticks on birds with ticks infected by Bbls [85]. Nevertheless, further observations are needed to confirm the vector competence of these ticks for *Borreliae* LG, focusing on the acquisition, maintenance, and subsequent transmission in a vertebrate host during the blood feeding [68].

### 3.3. Climate Change 

One of the consequences of global climate change, affecting habitat suitability and tick demographics, is its effect on infectious diseases and human health [86]. Global surface temperature has increased significantly over the past 50 years. Future climate scenarios include increased frequency and intensity of heat extremes, heavy precipitation, and agricultural and ecological drought. Pathogens, vectors, hosts, and disease transmission may be sensitive to these changing conditions. The reproduction and extrinsic incubation period of pathogens within a vector are temperature-dependent, and higher temperatures accelerate pathogen maturation. Environmental temperature influences the spatial and temporal distribution of disease vectors that transport and transmit pathogens to humans. Disease transmission may, in turn, be influenced by meteorological factors and by changes in contact between humans, competent animals, vectors, and pathogens [21].

Tick-Borne Relapsing Fever (TBRF), which is more common in central and southern Africa [87], can be vectored by *Ornithodors* spp. ticks, which thrive in low-humidity environments and desert areas (*Ornithodoros savignyi* vectors *Borrelia kalaharica*) [88]. *Ixodes* ticks, which carry Bbls, have a preference for humid environments. A humidity level of 84–98% is ideal for the survival of larvae, nymphs, adult ticks, and eggs [89]. *Ixodes* spp. ticks in northern endemic areas (Europe, North America) climb on the upper surface of leaves, grasses, and twigs waiting to attack hosts, whereas ticks in southern areas and Africa tend to remain under the surface of leaves [90]. Therefore, a summer walk in the woods can be related to direct exposure to *Ixodes* spp. in Europe and, to a lesser extent, in Africa. This is well correlated with the incidence of Lyme disease in humans [91].

### 3.4. Lizards as Reservoirs 

Differences in the spread of LB in Europe (North) and Africa (South) could be related to a dilution of the pathogen in Africa because of the different reservoirs (mammals and lizards). In Europe, *Ixodes* ticks mainly have rodents as reservoirs, while in Africa, they abound on lizards, which are known to be poor reservoirs of Bbsl [92]. Mice, voles, and shrews are usually excellent reservoirs for Bbsl, while larger mammals and lizards are not. Nevertheless, there are certain exceptions. It has been reported that the competence of lizards as reservoirs differs in Africa and America [41]. *Ixodes inopinatus*, indeed, infests the lizards *Psammodromus algirus*, *Lacerta agilis*, which are efficient reservoirs of Bbss in North Africa, amplifying its spread [65,93]. The host competence to transmit *Borreliae* LG was reported to vary between old-world and new-world lizards [41]. The old-world lizards (North Africa) seem to serve as amplifying hosts for *B. lusitaniae* and to be competent reservoirs in their maintenance and diffusion cycle [93]. New-world lizards (North America) seem to be poor hosts of Bbss, reducing the prevalence of this spirochete in nature. Studies along the West Coast of the United States have shown a consistent infestation of the fence lizards *Sceloporus occidentalis* by *Ixodes pacificus* ticks, which were minimally infected by Bbss [94,95].

### 3.5. LB in Humans, Domestic Animals, Livestock, and Wildlife 

The diagnosis of Lyme disease should be considered in patients in endemic areas who have had a tick bite and have clinical symptoms of LB. When erythema migrans is present, the diagnosis is clinical, without the need for laboratory tests of confirmation. Anti-*Borrelia* antibodies with a two-tiered test (ELISA and Western blot) are recommended when symptoms or clinical manifestations suggestive of LB are present [2].

In African countries, tick-borne zoonotic pathogens (TBDs) circulate widely. Their infections can affect humans, such as LB and Relapsing Fever (RF), and livestock, causing significant economic damage. Domestic livestock is, indeed, an integral part of many families in African countries. Therefore, for the prevention of these diseases, it is important to carry out surveillance and epidemiological studies of TBDs and LB in ticks, humans, animals, and reservoirs, combined with adequate laboratory diagnostics. Furthermore, it is necessary to increase border inspections of imported animals to stop the cross-movement of ticks and TBDs [54].

Personal protective measures, such as protective clothing and tick repellents, are recommended to prevent infection and transmission. During and immediately after exposure to a tick habitat, patients should be advised to examine their bodies and promptly remove attached ticks (statement of good practice). In African countries, these measures are often difficult to apply.

Regarding the therapy, the effectiveness of a short course of doxycycline (10 days) in the early phase of LB is welcome in African countries due to the low cost of the drug and the applicability of an effective treatment [96]. Nevertheless, doxycycline can cause photodermatitis, so amoxicillin is preferred during pregnancy [97]. In neuroborreliosis and in more severe forms, ceftriaxone iv can be used for 14–21 days [4].

Other factors, such as international travels, the transfer of pathogens between wild and domestic animals, and climate–environmental changes, can alter the spread and distribution of LB and its vectors [98]. Also, human activities, such as deforestation and urbanization, can impact the diffusion of tick-borne pathogens by damaging the vector habitat and increasing the possibilities of interaction between humans and animals [99].

## 4. Conclusions

In Africa, there is LB, but it is not endemic. At present, LB cases are limited to African countries bordering the Mediterranean Sea and the Indian Ocean, which have the proper climatic conditions and humidity for their survival. Bbls is transported in Africa by migratory birds infested by ticks of the *Ixodes* genus, which can infect humans, and also savanna animals, dogs, and horses. In addition, there is also the possibility of a tick switch from the *Ixodes* to the *Hyalomma* genus, as already shown [100]. Bbls reservoirs can be rodents, birds, and also lizards, which are relevant as possible amplifiers of LB in Africa. As a consequence, North African populations are vulnerable to LB as well as European ones to tick-borne pathogens characteristic of Africa. Climate change and migratory phenomena can further facilitate the mutual exchange of tick-borne pathogens from Europe to Africa and vice versa.

## Figures and Tables

**Table 1:** Lyme group *Borreliae* in Africa.

Countries	*Borreliae* Lyme Group	*Borreliae* LG Identified in			
		Human		Animals/Reservoir	Ticks
		Pos.	EM		
Algeria	*Borrelia lusitaniæ* (ID 3117), *B. garinii*, *B. burgdorferi* ss	Yes	Yes	*Turdus merula*, Lizards (*Psammodromus algirus*), Dogs, Horses (*Equus caballus*)	*Ixodes ricinus*, *I. inopinatus*
Morocco	*Borrelia lusitaniæ*, *B. garinii*, *B. burgdorferi* ss	Yes	Yes	*Turdus merula*, Lizards (*Psammodromus algirus*), Dogs, Horses	*Ixodes ricinus*, *I. inopinatus*
Tunisia	*Borrelia lusitaniæ* (genospecies Poti B2 and Poti B3), *B. garinii*, *B. burgdorferi* ss	Yes	Yes	*Turdus merula*, Lizards (*Psammodromus algirus*, *Lacerta agilis*), Dogs, Horses	*Ixodes ricinus*, *I. inopinatus*
Egypt	*Borrelia afzelii*, *B. burgdorferi* sl	Yes	Yes	Dogs, *Camelus dromedarius*	*Ixodes redikorzevi*, *I. ricinus*, *Hyalomma dromedarii*
Kenya	*B. garinii*, *B. burgdorferi* ss	Yes		Giraffe (*Giraffa Camelopardalis*)	*Hyalomma* *rufipes*
Mozambique	*Borrelia burgdorferi* sl	Yes		Rodents	*Hyalomma* spp.?
Tanzania	*B. burgdorferi* ss	Yes	Yes	Rodents	*Hyalomma* spp.?

Pos.: positivity; EM: erythema migrans.

## Data Availability

No new data were created or analyzed in this study. Data sharing is not applicable to this article.
